# Proteome profiling of enriched membrane-associated proteins unraveled a novel sophorose and cello-oligosaccharide transporter in *Trichoderma reesei*

**DOI:** 10.1186/s12934-023-02279-9

**Published:** 2024-01-16

**Authors:** Karoline Maria Vieira Nogueira, Vanessa Mendes, Karthik Shantharam Kamath, Anusha Cheruku, Letícia Harumi Oshiquiri, Renato Graciano de Paula, Claudia Carraro, Wellington Ramos Pedersoli, Lucas Matheus Soares Pereira, Luiz Carlos Vieira, Andrei Stecca Steindorff, Ardeshir Amirkhani, Matthew J. McKay, Helena Nevalainen, Mark P. Molloy, Roberto N. Silva

**Affiliations:** 1 Molecular Biotechnology Laboratory, Department of Biochemistry and Immunology, Ribeirao Preto Medical School (FMRP), University of Sao Paulo, Ribeirao Preto, SP Brazil; 2 Department of Natural Sciences, Macquarie University, Sydney, NSW Australia; 3 Australian Proteome Analysis Facility, Macquarie University, Sydney, NSW Australia; 4 Department of Physiological Sciences, Health Sciences Centre, Federal University of Espirito Santo, Vitoria, ES 29047-105 Brazil; 5 US Department of Energy Joint Genome Institute, Lawrence Berkeley National Laboratory, Berkeley, CA USA; 6 Department of Molecular and Cell Biology, Ribeirao Preto Medical School (FMRP), University of Sao Paulo, Ribeirao Preto, SP Brazil

**Keywords:** *Trichoderma reesei*, Cellulose, Membrane-associated proteome, Sugar transporters

## Abstract

**Background:**

*Trichoderma reesei* is an organism extensively used in the bioethanol industry, owing to its capability to produce enzymes capable of breaking down holocellulose into simple sugars. The uptake of carbohydrates generated from cellulose breakdown is crucial to induce the signaling cascade that triggers cellulase production. However, the sugar transporters involved in this process in *T. reesei* remain poorly identified and characterized.

**Results:**

To address this gap, this study used temporal membrane proteomics analysis to identify five known and nine putative sugar transporters that may be involved in cellulose degradation by *T. reesei*. Docking analysis pointed out potential ligands for the putative sugar transporter Tr44175. Further functional validation of this transporter was carried out in *Saccharomyces cerevisiae*. The results showed that Tr44175 transports a variety of sugar molecules, including cellobiose, cellotriose, cellotetraose, and sophorose.

**Conclusion:**

This study has unveiled a transporter Tr44175 capable of transporting cellobiose, cellotriose, cellotetraose, and sophorose. Our study represents the first inventory of *T. reesei* sugar transportome once exposed to cellulose, offering promising potential targets for strain engineering in the context of bioethanol production.

**Supplementary Information:**

The online version contains supplementary material available at 10.1186/s12934-023-02279-9.

## Background

*Trichoderma reesei* is a crucial platform for the industrial manufacturing of lignocellulolytic enzymes, which can be used to enzymatically break down one of the most abundant raw materials on the planet, the plant dry matter (lignocellulosic biomass (LB)). This process results in the production of economically important products, including biofuels and other bioproducts [1, 2].

Lignocellulosic biomass primarily comprises three components: lignin, hemicellulose, and cellulose [3]. Conversion of lignocellulosic biomass into biofuel such as ethanol—2nd generation ethanol (2G)—occurs through three main steps: pre-treatment, enzymatic hydrolysis, and fermentation [4]. In the enzymatic hydrolysis, the pretreated LB is broken down into soluble oligosaccharides [2]. *T. reesei* has a capacity to produce a diverse set of enzymes, including hemicellulases, cellulases and other Carbohydrate-Active Enzymes (CAZymes). These enzymes are potent players in carrying out the rapid enzymatic hydrolysis of LB [5].

During the process of holocellulose hydrolysis by *T. reesei*, a combination of monosaccharides and disaccharides released from LB function as regulatory agents of CAZymes encoding genes at the transcriptional level. Cellulose and other molecules, including sophorose and cellobiose, can trigger the induction of gene transcription, whereas glucose functions as an inhibitory carbon source, suppressing the expression of these genes [5–7]. Membrane proteins called sugar transporters are responsible for translocating these sugars across the cellular membrane. Several reports [8–12] have indicated the role of these proteins in cellulase regulation, either through transporting the inducer or repressor sugars into the cell or by sensing the extracellular cellulose. In this context, the manipulation of sugar transporters holds the potential to enhance cellulase production through two approaches: alleviating carbon catabolite repression (CCR) by deleting d-glucose transporters and improving inducer uptake by overexpressing transporters specific to substances like cellobiose or sophorose. This would be desirable, as the large-scale conversion of lignocellulose to ethanol represents an expensive process due to the high cost of enzymes such as cellulases [13]. Thus, knowledge of subsequent manipulation of *T. reesei* sugar transporters would offer a strategy to improve the economy of lignocellulose hydrolysis.

*Trichoderma reesei* boasts an effective membrane transportome, enabling it to effectively transport diverse carbon sources. In silico proteome analysis has estimated that *T. reesei* possesses approximately 100 sugar transporters [14, 15], although there is limited knowledge about their specific functions and roles in the cellulose degradation process.

Over the past years, there has been growing research on identifying novel putative sugar transporters in *T. reesei*. However, most of these analyses have relied on a combination of genome information and transcriptomic analyses conducted under various growth conditions. While these methods have provided valuable insights, they do not fully capture posttranscriptional regulatory events that can have a significant impact on protein abundance and localization. Therefore, proteomic analysis can offer a more accurate method for identifying the crucial sugar transporter components specific to a particular carbon source [16].

Our research aimed to obtain a broad view of the *T. reesei* sugar transportome during the process of cellulose breakdown. To accomplish this objective, we conducted a quantitative membrane proteome analysis of *T. reesei* under varying spatial conditions (carbon sources: cellulose and glycerol) and temporal conditions (culturing duration). Through this analysis, we generated an inventory of sugar transporters, including both known and previously unidentified ones, that might potentially have a function in sensing and transporting cellulosic hydrolysates. Significantly, our study identified a novel sugar transporter, Tr44175, capable of transporting cellobiose, cellotriose, cellotetraose, and sophorose.

This systemic data provides a foundation for a better understanding of *T. reesei* transportome and offers an opening avenue for advanced studies that aim to characterize and engineering well-known and new sugar transporters. Our work has the potential to contribute to the development of high-yielding cellulase strains of *T. reesei*, which could significantly improve the sustainable production of 2G ethanol.

## Results

### Potential sugar transporter candidates implicated in the cellulose degradation process by *T. reesei*

In this study, we aimed to identify the key sugar transporters present in the fungal cell membrane during a temporal analysis of the process of cellulose degradation by *T. reesei*. Our investigation focused on *T. reesei* cultures grown with glycerol or cellulose as carbon source. Glycerol was used as a control carbon source to elucidate the sugar transporters that become more abundant in fungal cells specifically in the presence of cellulose. The identified sugar transporters were analyzed to predict its topology and are summarized in Table 1. The False Discovery Rate (FDR) of peptide identification of less than 1% and high number of peptide-spectrum matches (PSMs) (4-222 PSMs) pointing back to each of the proteins indicates high confidence in the identification of the potential membrane proteins in our study (Table 1).

**Table 1:** Sugar transporters identified in this work by LC/MS analysis

**Protein ID**	**TMDs**	**pTM**	**Gravy values**	**Peptides**	**PSMs**	**Substrates**	**Refs.**
STR1 (ID 50894)	12	0.85	0.42	5	30	Glc, Fru, Gal, Man, Xyl, LAra	[17, 20, 21]
65153	12	0.88	0.38	9	48		
50618	12	0.75	0.15	18	213	Fru	[17]
CRT1 (ID 3405)	12	0.84	0.35	4	35	Glc, Sop, Cb, Lac	[18]
68812	12	0.84	0.23	12	222		
HXT1 (ID 22912)	12	0.82	0.21	5	20	Glc	[19]
STP1 (ID 47710)	12	0.83	0.37	11	149	Cb, Glc	[10]
44175	12	0.83	0.23	10	119	Cb, Sop, Clt, Cltr	This work
80875	12	0.81	0.45	7	39		
53903	12	0.89	0.39	2	5		
121850	12	0.82	0.25	2	4		
123702	12	0.75	0.42	6	64		
76800	12	0.89	0.31	9	111		
123809	12	0.79	0.70	2	10		

The Table provides information about the sugar transporters identified in this work by LC/MS analysis, including: the Gravy Value (GV), the number of transmembrane domains (TMDs), predicted template modeling scores (pTM) for the predicted structures, distinct peptide sequences identified in the protein group, and the number of peptide spectrum matches (PSMs). The protein IDs correspond to the sugar transporters identification according with JGI database. The FDR was less than 1%. Known substrates of the sugar transporters are indicated. LAra: l-arabinose; Clt: cellotriose; Cltr: cellotetraose; Gal: d-galactose; Fru: d-fructose; Xyl: d-xylose; Glc: d-glucose; Lac: lactose; Cb: cellobiose; Man: d-mannose; Sop: sophorose

Of the 14 sugar transporters we identified, five of them have previously been characterized: protein IDs 3405, 50894, 22912, 50618 and 47710. Among these, CRT1 (ID 3405), has been found to be related to cellulose sensing and cellulase induction [10]. It was shown to be necessary for the process of induction of cellulases by cellulose and lactose. STP1 (ID 47710) is another transporter that is capable of transporting both glucose and cellobiose. HXT1 (ID 22912), a glucose permease, has also been identified in *T. reesei* [19]. STR1 (ID 50894) is a crucial transporter for the utilization of pentoses by *T. reesei* [20]. Finally, the transporter Trire2_ 50618, known as FRT1, has been identified as capable of transporting fructose  [17]. This finding is of particular interest, as FRT1 has consistently demonstrated upregulation under cellulase-inducing conditions [23, 24].

**Fig. 1:**
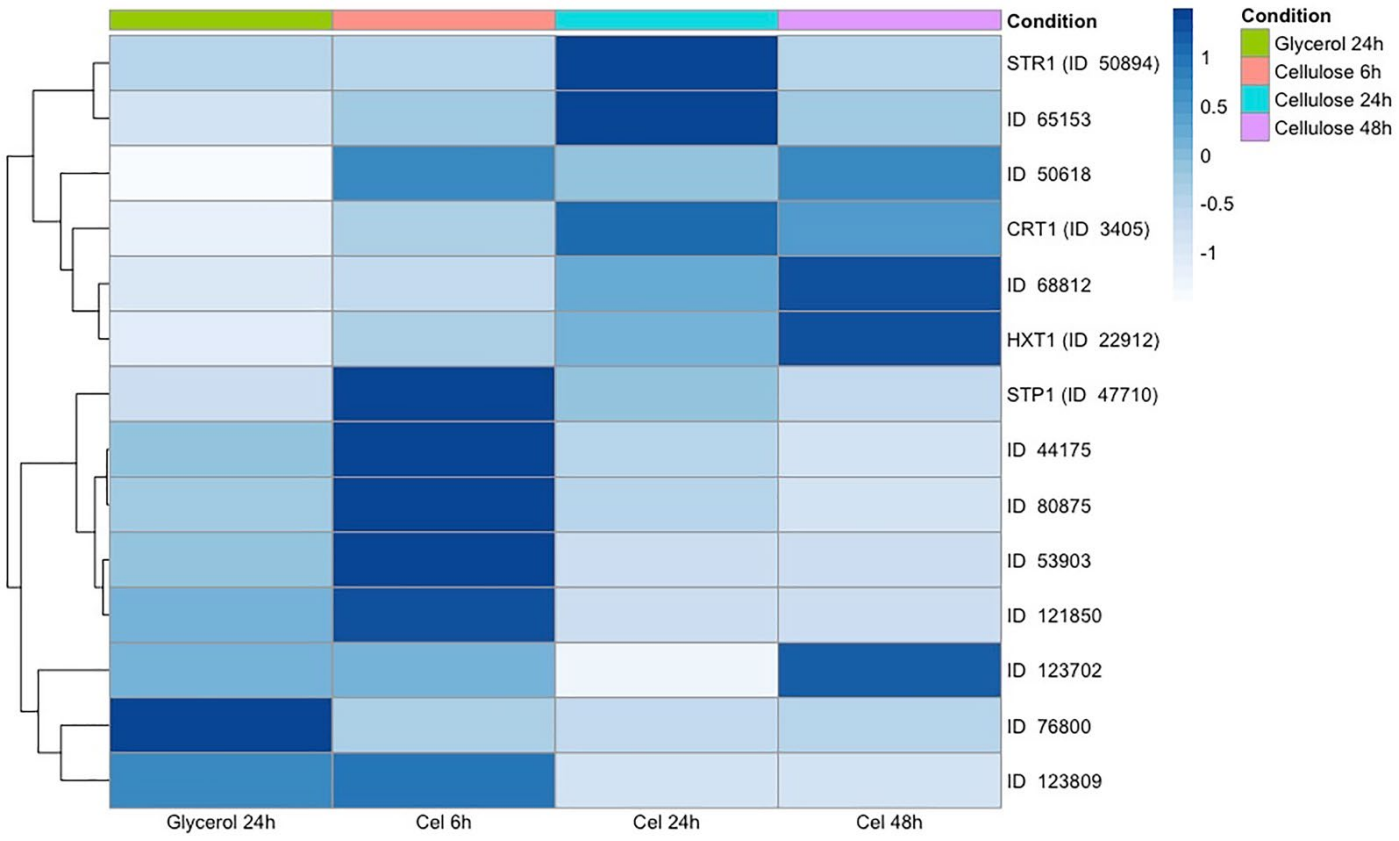
Abundance of the *T. reesei* sugar transporters in presence of glycerol and cellulose. The heat map represents the abundance of the sugar transporters identified during the culture of *T. reesei* in presence of glycerol and cellulose. The R heatmap package was utilized to perform hierarchical clustering. The hierarchical clustering of protein abundance in all conditions was carried out using the complete linkage method and Euclidean distance with row-centered values. The protein IDs correspond to the sugar transporters identification according with JGI database

Our findings revealed dynamic changes in the abundance of sugar transporters located in the cell membrane under various conditions. Specifically, we identified 12 sugar transporters that were present in all analyzed conditions. The protein abundance data displayed distinct profiles across the 6, 24, and 48-h culture periods on cellulose (Fig. 1). Notably, 10 sugar transporters exhibited less abundance when *T. reesei* was grown in the presence of glycerol. Conversely, these same proteins were more abundant in *T. reesei* cultures grown on cellulose (Fig. 1), in comparison to the glycerol condition. Intriguingly, the STP1 transporter (ID 47710) initially showed more abundance at 6 h of cellulose culture, followed by decreased abundance at 24 h. In contrast, the CRT1 transporter (ID 3405) demonstrated decreased abundance at 6 h when compared to 24 and 48 h of cellulose culture (Fig. 1). Additionally, five other putative MFS sugar transporters (protein IDs 80875, 123809, 121850, 53903, and 44175) exhibited upregulation at 6 h of culture.

### Phylogenetic analysis of identified sugar transporters

**Fig. 2:**
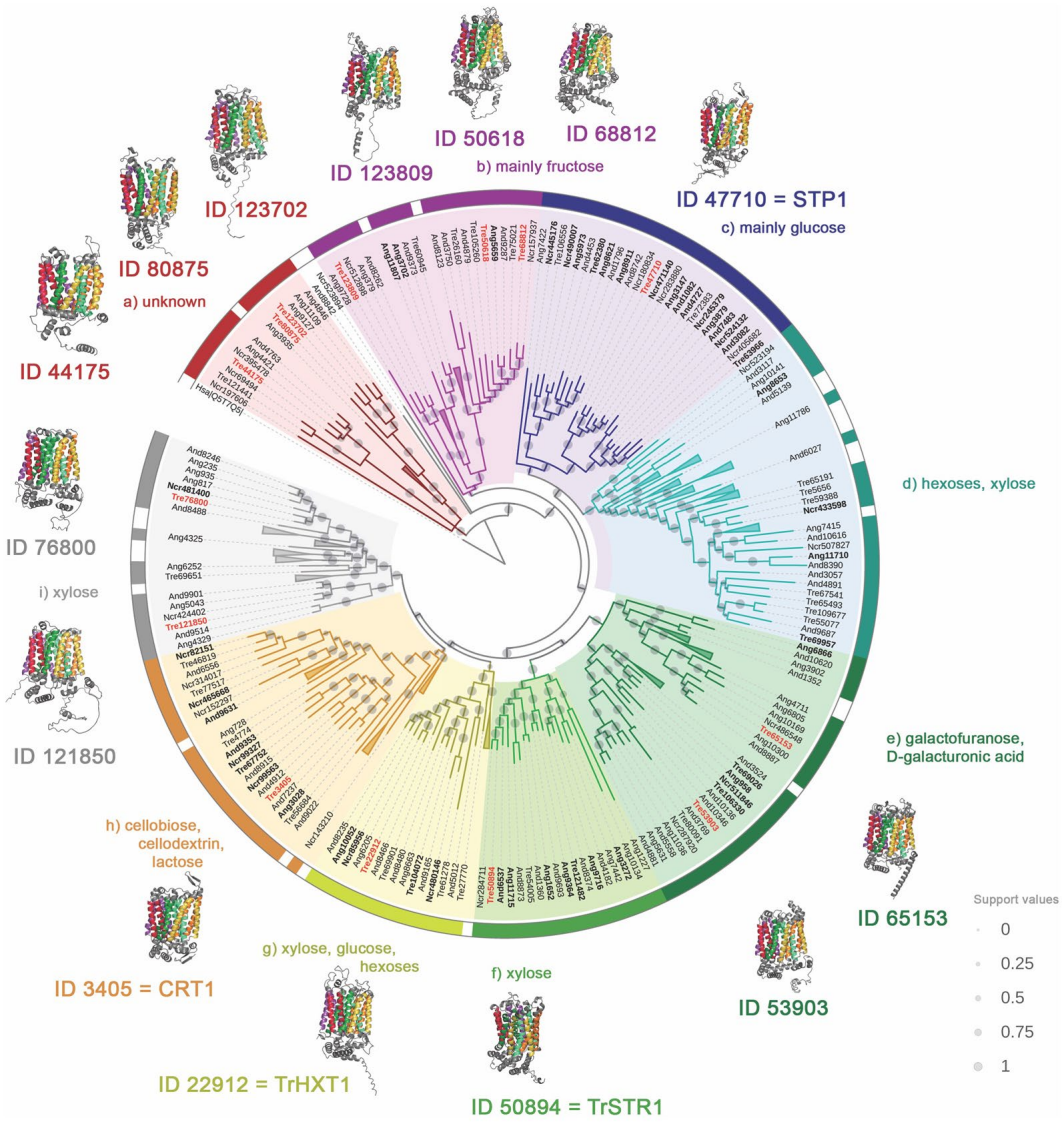
Sugar transporters subjected to phylogenetic classification. The tree was inferred using 335 protein sequences from *T. reesei*, *A. niger, A. nidulans* and *N. crassa* containing the Pfam PF00083 domain. The sugar transporters characterized experimentally in the literature [42] are highlighted with bold font and the transporters identified in this study are highlighted in red and bold. Each clade potential substrates are determined based on the functions of known sugar transporter members within those specific clades. Three clades in red did not resemble experimentally characterized sugar transporters. Support values from 1000 resamples are illustrated as gray dots

A phylogenetic analysis was conducted to determine the relatedness of the 14 potential sugar transporter proteins identified in this study to those of closely related, well-characterized fungi. In the phylogenetic analysis, among 333 sugar transporters described in Nogueira et al., 2020 [8]; 30 sugar transporters were already characterized. In addition, we have included 2 putative transporters identified in this study (protein IDs 80875 and 123702). Based on the biochemical properties described in the literature of the already characterized sugar transporters included in the phylogenetic tree, it was possible to attribute putative sugar specificity to each clade. The phylogenetic classification resulted in the division of transporters into 8 clades (A–I) (Fig. 2).

Figure 2 displays the structure of the sugar transporters identified in this study, also illustrating their distribution among various clades within the evolutionary tree. The 14 sugar transporters identified in this study are highlighted in red (Fig. 2), including those with known sugar specificity and unknown function. Of the identified transporters, five (protein IDs 50894, 3405, 47710, 22912, and 50618) are already characterized in *T. reesei.* For example, Clade C (Fig. 2) contains the STP1 transporter (47710), which can transport both glucose and cellobiose and is noted in a database as a high-affinity glucose transporter in *T. reesei.* Clade H (Fig. 2) contains the CRT1 transporter (ID 3405), which is found in the same cluster of cellobiose, lactose, and cellodextrin transporters already characterized. Still, in *T. reesei*, this transporter seems to be involved in the signaling process in the presence of cellulose rather than directly in cellobiose transport. Furthermore, through phylogenetic analysis, 3 sugar transporters identified in this study were found in clades with putative sugar transporters that have not yet been characterized (Fig. 2). Therefore, further functional analysis is necessary to establish the functions of these transporters.

### Expression analysis of *Tr44175* gene in *T. reesei* in response to cellulose or sophorose exposure

Among the sugar transporters examined in our study, we focused on transporter ID 44175 due to its distinct feature of high abundance in 6 h of cellulose exposure, as well as its increased expression in the presence of both cellulose and sophorose [24].

A previous study demonstrated a significant increase in CMCase activity after 2, 4, and 6 h of *T. reesei* growth in the presence of sophorose, whereas the same increase in CMCase activities on cellulose was only observed when the fungus was grown by 24, 48 and 72 h in the presence of this carbon source [24]. Castro et al. [5] also showed that sugar transporters are differentially expressed when *T. reesei* is grown in the presence of cellulose (24, 48, and, 72 h) or sophorose by 2, 4, and 6 h. Particularly, *Tr44175* exhibited 21- and 17-fold higher expression in the presence of cellulose and sophorose, compared to glucose, in the *T. reesei* QM9414 strain.

**Fig. 3:**
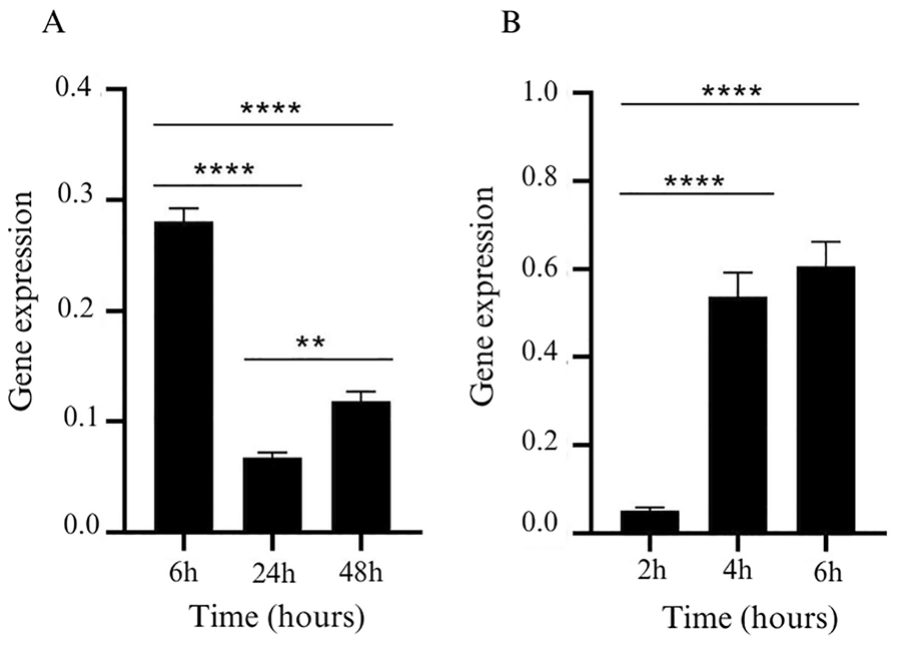
Expression analysis of *Tr44175* gene in *T. reesei*. The expression of the *Tr44175* gene was analyzed using *T. reesei* QM9414 strain grown in the presence of cellulose (**A**) or sophorose (**B**). The 2^–∆CT^ method [22] was used for calculating *Tr44175* gene expression relative to the endogenous control gene (actin)**.** Values are the means of three biological replicates. Data were analyzed using a 1-way ANOVA followed by a Bonferroni post hoc test. *P < 0.05; **P < 0.01; ***P < 0.001; ****P < 0.001

Considering these findings, we conducted a reverse-transcription quantitative real-time polymerase chain reaction (RT-qPCR) analysis to access the expression dynamics of *Tr44175* gene in the *T. reesei* QM9414 strain upon cellulose or sophorose exposure at specific time points. The culture times for sophorose induction were maintained as previously described by Castro et al. [5] while cellulose cultivation times were reduced up to 48 h to access the expression of *Tr44175* gene during the initial fungal response to the carbon source and correlate it with our proteomic data analysis. Our results revealed a noteworthy pattern: *Tr44175* expression increased at the 6-h time point, subsequently declining at 24 and 48 h, consistent with the proteomics data for cellulose (Fig. 3A). Notably, when grown in the presence of sophorose, *Tr44175* exhibited robust expression at 4 and 6 h (Fig. 3B). During 6 h of culture, the absolute expression of *Tr44175* in sophorose conditions was approximately 2.1-fold higher compared to its expression when *T. reesei* was cultured in cellulose for the same duration.

### In silico analysis: molecular docking by energy minimization

**Fig. 4:**
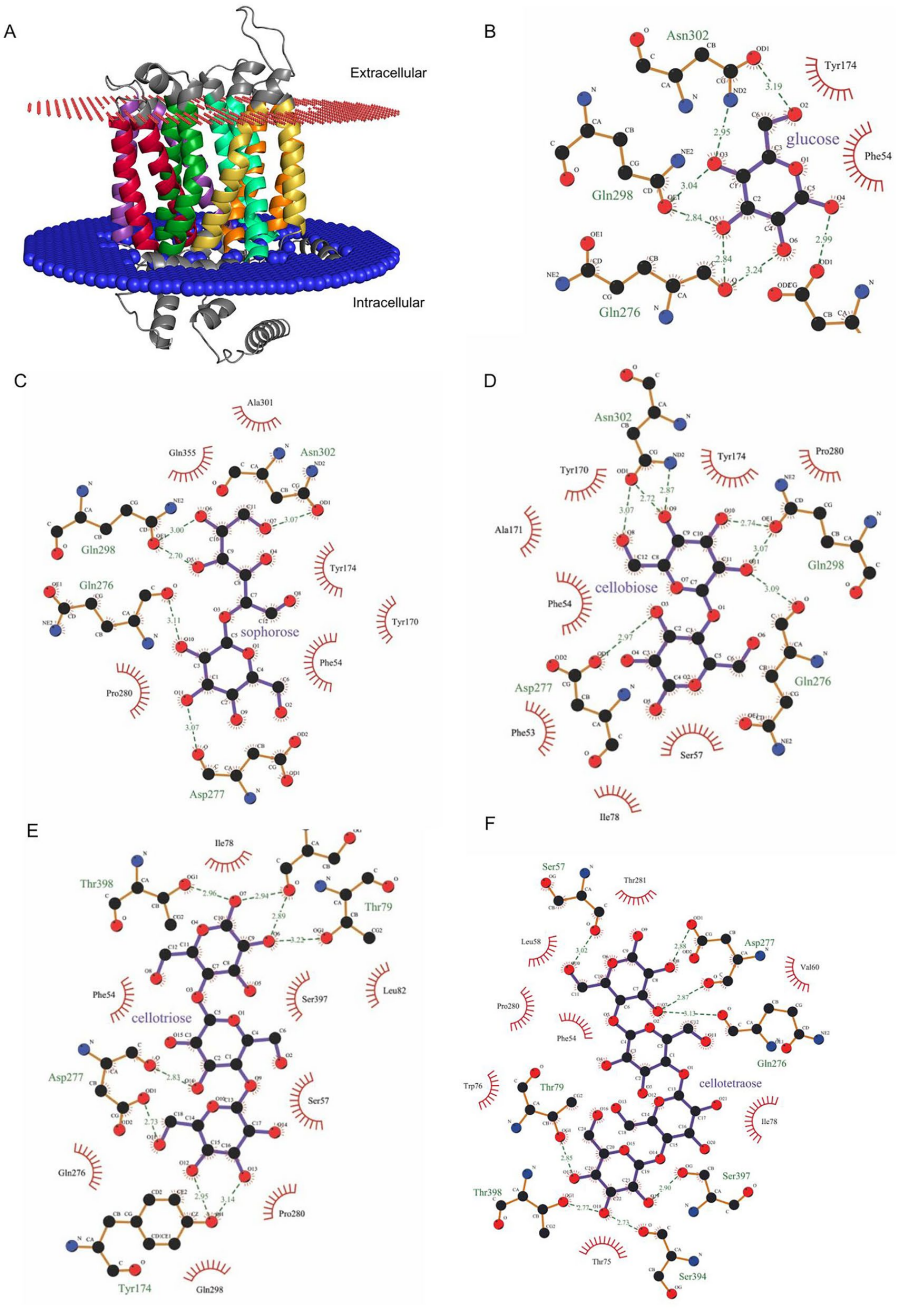
Prediction of membrane protein topology and docking analysis. **A** Analyses of membrane protein topology and insertion energy were performed in QMEANBrane. Interactions between Tr44175 and glucose (**B**), sophorose (**C**), cellobiose (**D**), cellotriose (**E**) and cellotetraose (**F**) analyzed using LigPlot + v2.2.8

To predict the binding capacity between Tr44175 and potential binding sugars derived from the biomass hydrolysate, we performed molecular docking analysis. We included glucose, cellobiose, cellotriose, cellotetraose, and sophorose as potential ligands. The energy minimization model and simulated membrane insertion energy model of Tr44175 protein revealed its topology with 12 transmembrane segments (Fig. 4).

The molecular docking analysis based on total free energy indicated that the coupling between Tr44175 and all analyzed sugars was energetically favorable (Table 2 and Fig. 4). The data also showed that this sugar transporter had a greater binding affinity for oligosaccharides and sophorose than for glucose (Table 2). The specific amino acid residues involved in the interactions with the analyzed ligands were also identified Table 2).

**Table 2:** Binding free energy (kcal/mol) of protein/ligand complexes and amino acids residues interacting with the ligand

Ligand	Binding free energy Tr44175_ligand complex	Amino acids residues interacting with the ligand
Glucose	− 5,5	Phe54, Tyr174, Gln276, Asp277, Gln298, Asn302
Cellobiose	− 6,1	Phe53, Phe54, Ser57, Ile78, Tyr170, Ala171, Tyr174, Pro280, Gln276, Asp277, Gln298, Asn302
Cellotriose	− 7,1	Phe54, Ser57, Ile78, Leu82, Gln276, Pro280, Gln298, Ser397, Tyr174, Asp277, Ser394, Thr398
Cellotetraose	− 7,2	Phe54, Leu58, Val60, Thr75, Trp76, Ile78, Tyr174, Pro280, Thr281, Ser57, Thr79, Gln276, Asp277, Ser394, Ser397, Thr398
Sophorose	− 6,4	Phe54, Tyr170, Tyr174, Pro280, Ala301, Gln355, Gln276, Asp277, Gln298, Asn302

Importantly, our data suggest that Tr44175 has the potential to transport diverse sugars, including cellobiose, sophorose, and glucose. Notably, sophorose is a strong inducer of cellulases in *T. reesei* [24, 25], and transporters for this disaccharide in this fungus have not been well-characterized in the literature. The promising interaction observed between Tr44175 and various sugars indicates the protein's ability to adjust to diverse carbon sources that may be present in the hydrolysate.

In summary, the molecular docking analysis of *T. reesei* Tr44175 protein supports its potential to transport diverse sugars, with a stronger binding affinity for disaccharides such as cellobiose and sophorose. These findings provided important information for further in vivo protein–sugar transporting analysis.

### Functional validation of Tr44175

To validate the transporter capacity of the putative *T. reesei* sophorose and cello-oligosaccharide transporter identified in this study (Tr44175), *S. cerevisiae* strain SC9721 was genetically modified to metabolize sophorose, cellobiose, cellotriose, and cellotetraose as the only carbon source. As *S. cerevisiae* is not capable of transporting and metabolizing cellodextrins and sophorose, it served as a null transporter system for this study.

**Fig. 5:**
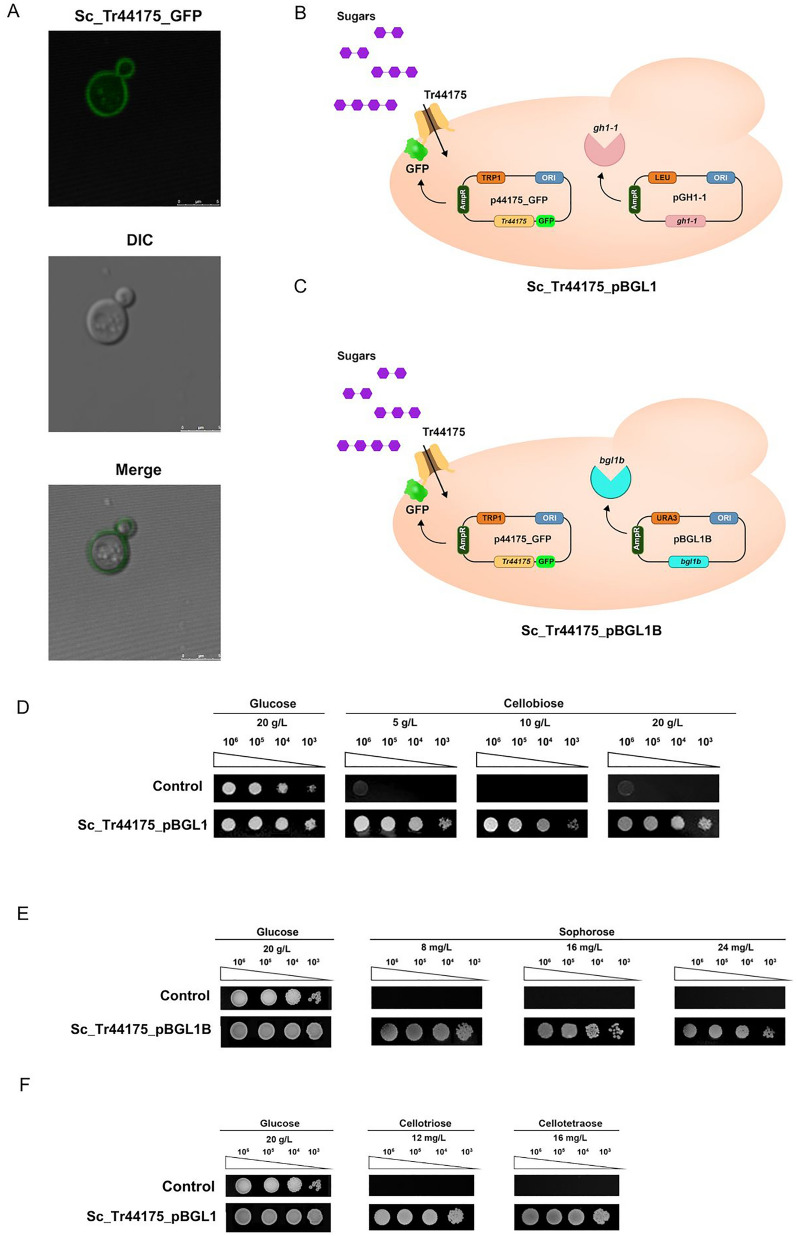
Tr4475 functional characterization. **A** Confocal microscopy shows that Tr44175::GFP localizes to the *S. cerevisiae* plasma membrane. Confocal microscopy pictures were taken in both differential interference contrast (DIC) and fluorescence modes and then merged (Tr44175::GFP). **B** Design of Sc_Tr44175_BGL1 cells with a vector expressing the transporter fused with GFP and a vector carrying a β-glucosidase encoding gene *gh1-1* from *N. crassa.* **C** Design of Sc_Tr44175_BGL1B cells with a vector carrying the transporter fused with GFP and a vector expressing a β-glucosidase encoding gene An03g03740 (designated *bgl1B*) from *A. niger.* **D** Growth of *S. cerevisiae* strain Sc_Tr44175_BGL1in YNB supplemented with 5 g/L, 10 g/L, or 20 g/L cellobiose concentrations. **E** Growth of *S. cerevisiae* strain Sc_Tr44175_BGL1B in the presence of 8 mg/L, 16 mg/L, and 24 mg/L of sophorose concentrations. **F** Growth of *S. cerevisiae* strain Sc_Tr44175_BGL1 in YNB supplemented with 12 mg/L of cellotriose or 16 mg/L of cellotetraose concentrations. The control strain contains the pRH195d empty vector and the gene encoding β-glucosidase GH1-1 or BGL1B. Yeast cells, when inoculated on media containing glucose or cellobiose, were incubated at 30 °C for 120 h, while those on media containing cellotriose, cellotetraose, or sophorose were incubated for 192 h

*Saccharomyces cerevisiae* SC9721 was engineered to express the *T. reesei* Tr44715 protein. To determine the location of the Tr44175 transporter into the cell, the gene coding to *Tr44175* transporter was fused to the gene encoding the GFP and observed its subcellular localization using confocal microscopy. The resulting Sc_Tr44175_GFP strain showed a fluorescence halo at the cell borderline, confirming the localization of the Tr44175 transporter in the yeast cell membrane (Fig. 5A).

Further, the pGH1-1 plasmid, containing the β-glucosidase-encoding gene *gh1-1* from *Neurospora crassa*, or the pBGL1B plasmid carrying the An03g03740 gene (*bgl1B*) [26] which encodes a β-glucosidase from *Aspergillus niger*, was introduced into the Sc_Tr44175_GFP strain (Fig. 5C). Positive transformants carrying the specific β-glucosidases and Tr44175 were isolated, and their ability to grow in the presence of cellobiose, cellotriose, cellotetraose, and sophorose was examined. Glucose was used as a growth control, and a transformant carrying pGH1-1 or pBGL1B and an empty vector was used as negative control.

After an incubation period of 120 h in the presence of cellobiose and 192 h in the presence of sophorose, cellotriose, and cellotetraose, the expression of Tr44175 restored the growth of the Sc_BGL1 or Sc_BGL1B strain in all sugars evaluated (Fig. 5D–F). The transporter Tr44175 allowed the engineered strains to grow in the presence of cellobiose, cellotriose, cellotetraose, and sophorose (Fig. 5), but no growth was observed in the presence of glucose when this transporter was expressed in a null *S. cerevisiae* strain (EBY.VW.4000) (Additional file 1: Fig. S1). These results indicate that the transporter Tr44175 is functional and capable of transporting cellobiose, cellotriose, and cellotetraose, as well as sophorose, a potent cellulase inducer.

**Fig. 6:**
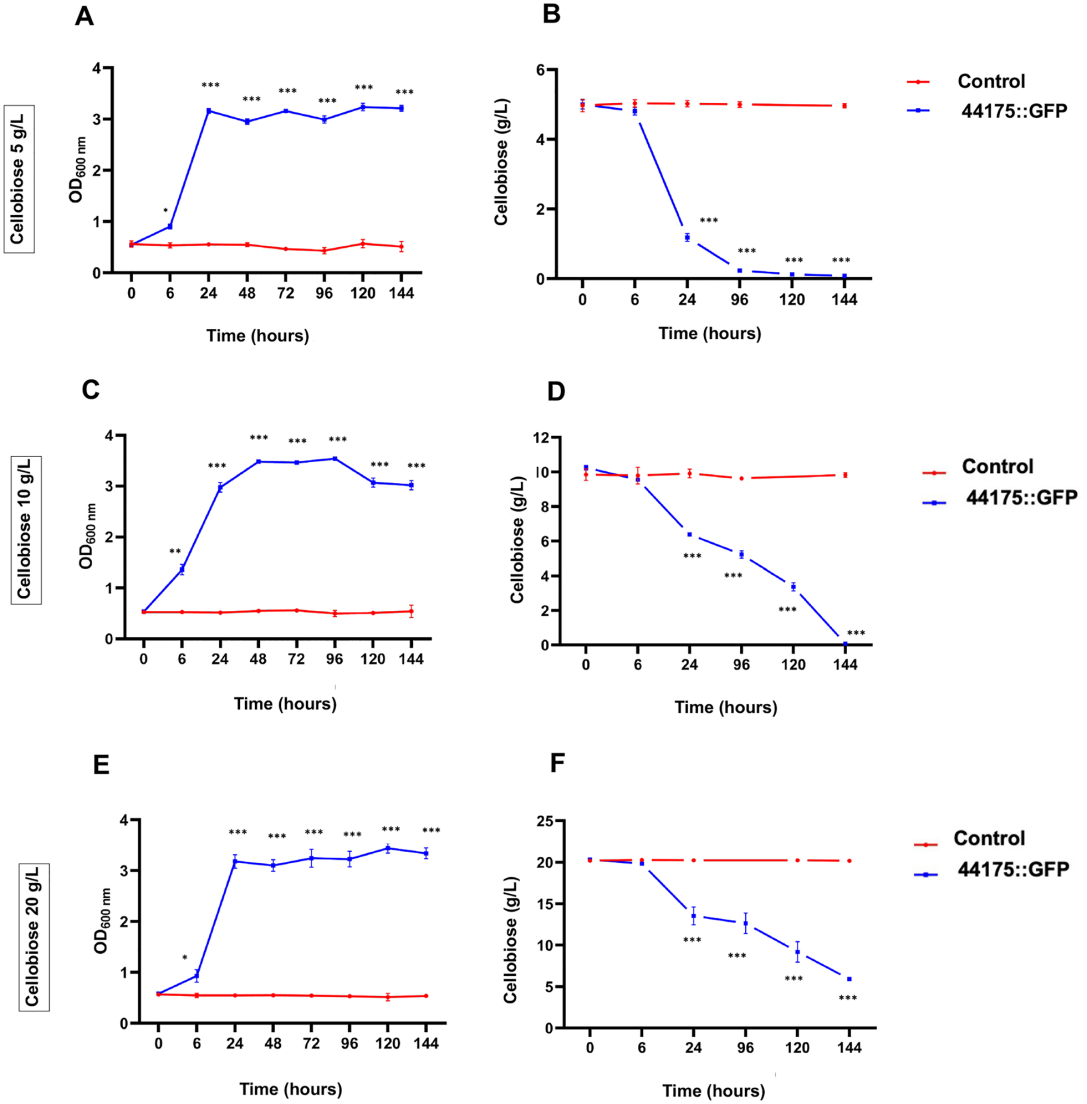
Growth and cellobiose consumption of *S. cerevisiae* strains expressing Tr44175. Growth of Sc_Tr44175_BGL1 (44175::GFP) and Sc_pRH195d_BGL1 (control), as determined by optical density at 600 nm (OD_600nm_) in YNB supplemented with 5 g/L cellobiose (**A**); 10 g/L cellobiose (**C**) or 20 g/L cellobiose (**E**). Cellobiose consumption of the Sc_Tr44175_BGL1 (44175::GFP) and Sc_pRH195d_BGL1 (control) when incubated in YNB supplemented with cellobiose 5 g/L (**B**); 10 g/L cellobiose (**D**) or 20 g/L cellobiose (**F**). Data were analyzed using a 1-way ANOVA followed by a Tukey post hoc test. *P *P < 0.01; **P < 0.001; ***P < 0.001

To conduct a more thorough analysis of Tr44175 transporter capacity and to confirm the spot-assay results, Sc_Tr44175_BGL1 strain was grown in cellobiose for further experiments. Growth curves of the Sc_Tr44175_BGL1 strain indicate that the expression of genes encoding *Tr44175* and the *gh1-1* β-glucosidase in SC9721 supported growth in different concentrations of cellobiose (5 g/L, 10 g/L, and 20 g/L), showing a slightly increased growth pattern at a concentration of 10 g/L (see Fig. 6C). However, a similar growth pattern was observed when analyzing lower concentrations (5 g/L) (see Fig. 6A) and higher concentrations (20 g/L) (see Fig. 6E). Cellobiose consumption was also confirmed in yeast growth in liquid media. After 144 h of culture, Tr44175 allowed for approximately 98.4% cellobiose consumption at lower concentrations (5 g/L) (see Fig. 6B), 99.2% at intermediary concentrations (10 g/L) (see Fig. 6D), and 70.5% at higher concentrations (20 g/L) (see Fig. 6F). These data suggest a potential inhibition of this sugar transporter in the presence of higher concentrations of cellobiose.

## Discussion

Several studies have made significant contributions to the identification of new sugar transporters in *T. reesei* through transcriptomes and proteomics analysis [23, 24, 27, 28]. Still, limited research has focused on investigating the role of sugar transporters during biomass degradation in this fungus. Oligosaccharides derived from the breakdown of cellulose components are pivotal in the induction of cellulase expression [29]. The uptake of these cello-oligosaccharides, resulting from cellulose hydrolysis, acts as a potential control point for activating cellulolytic genes [8, 30]. Certain sugars, transported into fungal cells through membrane proteins, can either repress (e.g., glucose) or induce (e.g., cellobiose and sophorose) the expression of xylanase and cellulase genes [24]. Transcription factors and regulatory molecules play a role in governing the expression of sugar transporters in *T. reesei* during sugar uptake [14, 31, 32]. Previously, the Tr44175 transporter was identified as upregulated in the presence of cellulose and sophorose [5]. Furthermore, a transcriptome analysis of a *T. reesei* strain, lacking a positive regulator of CAZyme expression, revealed the strong modulation of the Tr44175 transporter by XYR1 [14]. In this current study, Tr44175 shows to be more abundant in fungal cell membranes in the first six hours of culture. This work, in conjunction with these previous findings, highlights the potential involvement of the Tr44175 transporter in cellulose degradation in *T. reesei*. Furthermore, in vivo functional studies of Tr44175 have provided valuable insights into its capability to transport various cello-oligosaccharides derived from cellulose, such as cellobiose, cellotriose, cellotetraose, and sophorose.

**Fig. 7:**
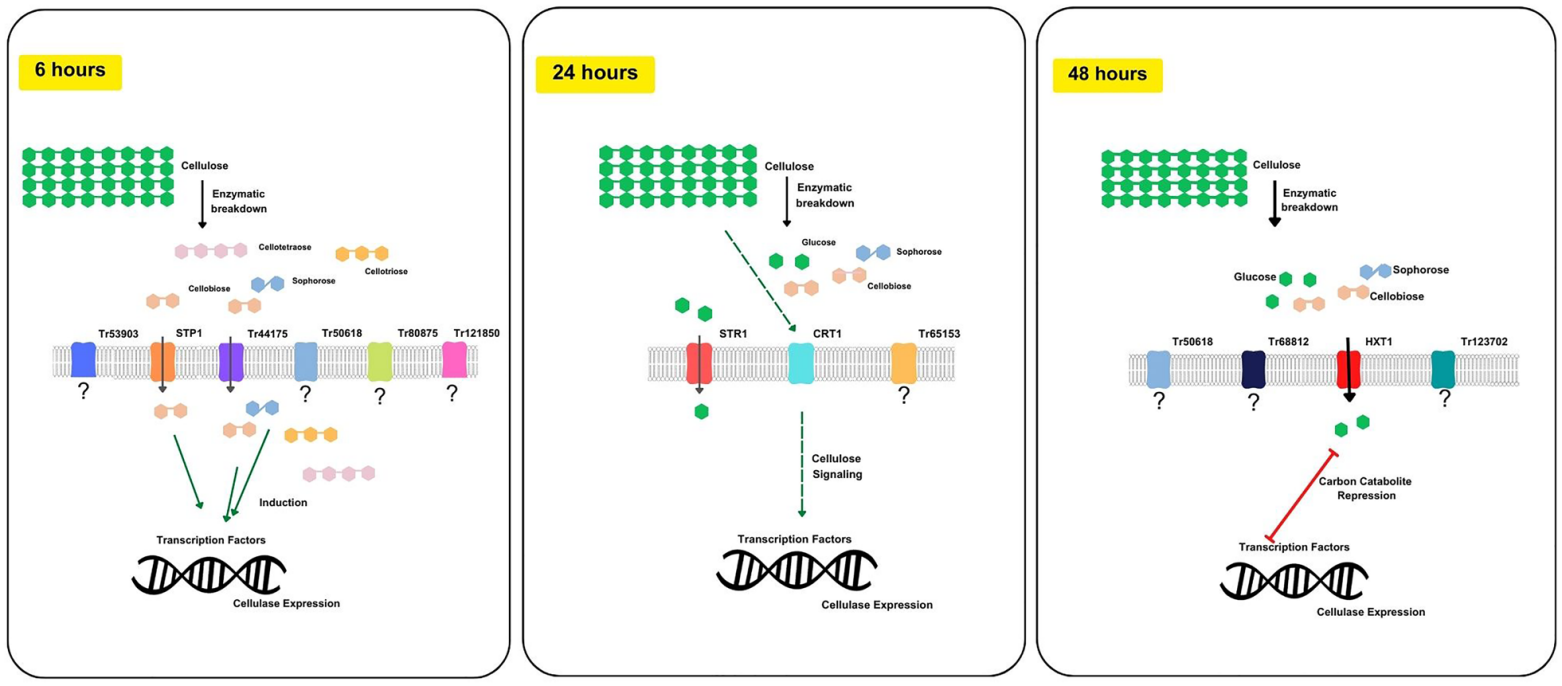
Inventory of *T. reesei* Sugar Transportome in the Presence of Cellulose. A hypothesized model of sugar transporters-mediated cellulase induction in *T. reesei*. The figure presents an overview of the potential transport activity and cellulose signaling by known sugar transporters and their suggested role in cellulase induction. Besides that, this scheme shows all the sugar transporters identified in this study, including the uncharacterized ones

By conducting a systematic analysis, in this work, a total of 14 sugar transporters were identified, with 5 being previously characterized in *T. reesei.* The abundance of these transporters was found to vary at different time points, indicating their importance during specific time intervals in the transportome network and the process of cellulase induction during cellulose breakdown. Based on the results obtained in this study, a hypothesis regarding the function of the major sugar transporters identified at various time points has been postulated (Fig. 7).

Although cellulose is a solid substrate and can trigger cellulase induction, a soluble product released from cellulose via basal *T. reesei* cellulase activity is considered the actual inducer. It is plausible that during the first 6 h of culture, STP1 and Tr44175 could initiate the cascade of cellulase induction by transporting small amounts of oligosaccharides that are generated from the basal cellulase activity [33]. Interestingly, during this period, seven sugar transporters were detected to be present in the fungal cell membrane, likely indicating the potential actuation of these proteins in the initial signaling cascade. After induction by these sugars, in 24 h other transporters are translocated to the fungal cell membrane, and CRT1 signal the presence of cellulose, resulting in higher rates of cellulases in the media and of mono-disaccharide uptake. STR1 initiates glucose uptake at very low concentrations, while an unknown transporter Tr65153 (ID 65153) also acts during this interval of time. In 48 h, HXT1 is potentially translocated to the membrane, maintaining glucose uptake at low concentration while other potential glucose transporters are also translocated to the fungal cell membrane. Accumulation of glucose inside the cell causes the carbon catabolite repression and cellulase production is limited. Interestingly, a *T. reesei* cellobiose transporter previously reported by our group, Tr69957 [12], the cellobiose transporter Tr_StrC (Trire2_67752) [34] and the transporter-like protein CRT2 that is reported to potentially acts as a signal transductor in cellulase induction [35] were not identified in this work, which could be explained by low abundance or by regulatory events at the post-transcriptional level.

The systematic approach applied in this work has provided an understanding of the sugar transportome in *T. reesei*, obtaining a broad overview of the most significant sugar transporters that are potentially involved in cellulose degradation in this fungus. *T. reesei* may employ a sophisticated mechanism to detect changes in its environment, allowing it to respond precisely to sugar availability and regulate sugar uptake and cellulase gene expression through multiple signaling pathways.

## Conclusion

The present study has successfully identified key sugar transporters that potentially play a crucial role in the process of cellulose degradation, expanding the potential for discovering new sugar transporter candidates. However, further research is necessary to fully understand their involvement in cellulase gene expression. Additionally, this study has unveiled a transporter capable of transporting cellobiose, cellotriose, cellotetraose, and sophorose. These findings present exciting prospects for future strain engineering endeavors. In conclusion, this study highlights the importance of conducting temporal analyses of sugar transporters to determine their significance at specific time intervals and their impact on cellulase induction during cellulose degradation. Such investigations can provide valuable insights into optimizing cellulolytic processes and improving the efficiency of cellulase production.

## Methods

### Fungal growth conditions

The *T. reesei* QM9414 (ATCC 26921) strain was cultured on MEX medium (consisting of 2% agar and 3% malt extract) at a temperature of 28 °C until complete sporulation. For proteomic analysis, QM9414 strain was cultivated on PDA (Potato Dextrose Agar medium) and spore suspension of approximately 10^6^ mL^−1^ was inoculated into 1L flask containing 200 mL of Mandels-Andreotti medium (MAM) (pH 5) supplemented with microcrystalline cellulose (MCC) (1% (w/v)) or glycerol (1% (w/v)). For cellulose culture, 24 h glycerol culture was subsequently filtered, and the mycelia obtained was transferred to a fresh 200 mL MAM supplemented with 1% (w/v) MCC. This process was conducted according to Castro et al. [5]. The cultures containing 1% MCC were subjected to incubation on an orbital shaker set at 200 rpm, maintained at 28 °C for durations of 6, 24, and 48 hours. Three biological replicates were performed for all experiments. After filtration, the obtained mycelia were promptly frozen using liquid nitrogen. For further protein extraction, the frozen mycelia were stored at − 80 °C.

### Enrichment of membrane-associated proteins

Membrane-associated proteins were purified following a previously established protocol [16] with modifications. In summary, frozen mycelium samples (2–3 g, press-dried) from fungal cultures grown for 6, 24, and 48 h were mechanically disrupted by griding using the mortar and pestle tools while immersed in liquid nitrogen. The resulting material was then resuspended in a 20 mM HEPES buffer with a pH of 7.6, supplemented with cOmplete™ EDTA-free Protease Inhibitor Cocktail (Roche), and 150 mM NaCl. Subsequently, the samples underwent a centrifugation step at 500×*g* for 5 min. The resulting supernatant was collected and further centrifuged at 5000×*g* for 20 min. The remaining supernatants were subjected to a final centrifugation step (~ 85,000×*g*) for 120 min using a Beckman Coulter Optima XPN centrifuge. The obtained precipitate was resuspended in a solution containing 100 mM of triethylammonium bicarbonate and 1% sodium deoxycholate detergent. This resuspended mixture was vigorously mixed using a vortex mixer and incubated in a thermomixer at 95 °C and 800×*g* for 5 min. The Pierce™ BCA Protein Assay Kit was used to quantify the protein associated content of the plasma membrane fraction.

### LC–MS/MS sample preparation

#### In-solution digestion

Each protein sample, containing an equal amount (100 µg), underwent a reduction step using 10 mM DTT (dithiothreitol) for 30 min at 60 °C. Right after, alkylation was performed using 20 mM IAA (iodoacetamide) for 30 min at room temperature. Following this, 5 mM DTT was added, and the samples were incubated for 15 min at room temperature. Finally, trypsin digestion of the proteins was carried out at a protein-to-enzyme ratio of 50:1, at 37 °C overnight.

#### Mass spectrometric measurements

The LC–MS/MS data acquisition was accomplished using a Thermo Fisher Scientific Q-Exactive-HF-X mass spectrometer coupled with a Dionex Ultimate 3000 nRSLC nanoliquid chromatography system. The process involved the separation of proteolytic peptides using a C18 reverse-phase precolumn (*in-house* packed, halo-C18, 100 μm inner diameter, 2.7 μm particle diameter, 3.5 cm in length) connected to a C18 reverse-phase analytical column (*in-house* packed, halo-C18, 15 cm in length, 75 μm inner diameter, and 2.7 μm particle diameter). Peptide elution was carried out over a 130-min gradient, starting from 2% and increased up to 30% acetonitrile in ddH_2_O with 0.1% formic acid, at a flow rate of 300 nL/min. The eluted peptides were electrosprayed into the Q-Exactive-HF-X mass spectrometer for analysis.

An electrospray voltage of 2.6 kV was applied, and peptide precursors with an m/z range of 350 to 1400 were scanned at a resolution of 60,000 with an AGC (Automatic Gain Control) target value of 3 × 10^6^. For fragmentation, the top 20 ions with the highest intensity from the previous survey scan were subjected to Higher-Energy Collisional Dissociation (HCD) at normalized collision energy of 28 and a 1.3 m/z isolation width. The MS method utilized a minimum signal threshold of 4 × 10^3^ for MS2 triggering, an AGC target value of 2 × 10^5^ for MS2, and a maximum injection time of 60 ms for MS2. Only precursors with a charge state ranging from + 2 to + 6 were subjected to MS/MS analysis with the MS/MS scan resolution set at 15,000. To avoid selecting previously fragmented ions, a dynamic exclusion time of 90 s was implemented. Furthermore, after each sample injection, a blank injection was performed to minimize the presence of carryover proteins.

### Analyzing proteomics data

The LC–MS/MS spectra’s acquired from the proteomics experiment were processed and analyzed using Proteome Discoverer v2.2 software (Thermo Fisher Scientific) with the SEQUEST algorithm. The *T. reesei *in silico proteome acquired from the JGI database peptides (https://genome.jgi.doe.gov/Trire2/Trire2.home.html) was used for peptides mapping. Before the main search, offline mass accuracy recalibration was performed using a spectral recalibration node. The database search allowed for up to two missed cleavages, with cysteine carbamidomethylation of cystine set as a static modification. Fragment mass and the precursor tolerances were set at 0.02 Da and 10 ppm, respectively. Dynamic modifications included, deamination (N and Q), acetylation of NH2-terminal methionine, Gln- > pyro-Glu (Q, peptide-terminus), oxidation (M), and acetylation of NH2-terminus with methionine loss (Protein N-terminus). False discovery rate (FDR) was calculated using Percolator with reverse sequence database searches and an FDR threshold of 1%.

For label-free MS1 quantification, the Minora feature detector algorithm was used to identify chromatographic peaks. The feature mapper node was employed to map features across multiple files with specific settings (maximum retention time shift of 15 min, a minimum signal-to noise threshold of 4, and 6-ppm mass tolerance). Peptides with certain modifications (deamination (N, Q), Gln- > pyro-Glu (Q, peptide N-terminus), acetylation of NH2 -terminus with methionine loss (Protein N-terminus)) were excluded in protein quantification analysis. Protein area values were exported and analyzed using Excel. The Perseus software platform was utilized for protein abundance analysis. Proteins present in at least two out of three replicates were considered, and missing values were imputed using the imputation function in Perseus. A t-test was performed to compare protein quantities across different conditions, with criteria for statistical significance defined as a two-fold change in abundance and a t-test p-value less than 0.05.

### Phylogenetic tree construction

To construct the phylogenetic tree, we used the amino acid sequences from 333 transporters presented in the literature [8] as well as 2 transporters identified in this work (IDs: 80875 and 123702). Identification and reference of all the proteins used to construct the phylogenetic tree can be found in the Additional file 2: Table S1. These sequences correspond to sugar transporters produced by the fungi *Aspergillus nidulans*—112, *Aspergillus niger*—114, *Neurospora crassa*—43, and *Trichoderma reesei*—66; and all of them were obtained from JGI Genome Portal [36, 37]. MAFFT [38] was used to perform the multiple sequence alignment according to the default parameters. The full sequences were used to perform the analysis. Next, the phylogenetic tree was inferred using FastTree 2 [39]. The visualization of the results was constructed using iTool [40].

### Prediction of protein structure and molecular docking

To predict the structure of the protein 44175, the corresponding amino acid sequence was obtained from the *Trichoderma reesei* v2.0 genome, available from the Joint Genome Institute (JGI) [36]. Using the ColabFold implementation [41] of Alphafold2 [42], 5 models were generated. The evaluation of the predictions was carried out using the PROCHECK [43], QMEANBrane [44], ERRAT [45], and ProSA-Web [46]. The chosen model was used for molecular docking analysis using AutoDock Tools [47] for the preparation of the model and the ligands and AutoDock Vina [48] for docking between them. The PubChem [49] database was used to obtain the ligands (cellobiose CID: 10712, cellotetraose CID: 439626, cellotriose CID: 5287993, glucose CID, and sophorose CID: 92797) used for docking analysis. The validation of the process was performed through the redocking of 3 complexes available in PDB [50]. In all the protocols, the docking was not directed towards any specific part of the protein (blind docking). Interactions between the proteins under study and the ligands were analyzed using LigPlot + v2.2.8 [51].

### Membrane proteins prediction

The PPM 3.0 [52] and DeepTMHMM [53] algorithms were employed to predict transmembrane domains. The Gravy values, as described by Kyte et al. in 1982 [54], were determined by utilizing the Sequence Manipulation Suite's Protein GRAVY score calculator tool, accessible at http://www.bioinformatics.org/sms2/protein_gravy.html.

### RT-qPCR analysis

For *Tr44175* gene expression analysis, 10^6^ spores/mL of QM9414 were precultured in Mandels–Andreotti medium (MAM) containing 1% (v/v) glycerol at 30 °C for 24 h. Mycelia were filtered with Miracloth (Merck) and washed twice with MAM without any carbon source. The mycelium was then transferred to a MAM medium containing 1% Avicel or 0.5 mM sophorose as the sole carbon source. Cultures were inoculated at 30 °C for 6, 24, and 48 h in Avicel and 2, 4, and 6 h in Sophorose within a shaker operating at 200 rpm. All mycelia were collected and stored at − 80 °C for total RNA extraction. The experiments were performed in three biological replicates. Total RNA was extracted using the TRI Reagent® (Sigma-Aldrish) according to the manufacturer’s instructions. RNA integrity was confirmed by electrophoresis analysis in 1% (m/v) agarose. 1 µg of total RNA was digested with DNAse (Invitrogen) to remove gDNA. Reverse transcription of the RNA template was performed using the Maxima First Strand cDNA Synthesis Kit for RT-qPCR (Thermo Scientific™) according to the manufacturer’s instructions. Quantitative PCR was performed using the SsoFast™ EvaGreen® Supermix (Bio-Rad) on a Bio-Rad CFX96™ thermal cycler. Data analysis was performed using the threshold cycle according to the 2^−ΔCT^ method, normalized toactin transcript levels [22]. The experiment was performed with three biological replicates.

### In vivo functional characterization

#### Construction of engineered yeast strains

The *S. cerevisiae* SC9721 strain served as the background strain for constructing yeast strains expressing the Tr44175 transporter and specific β-glucosidases. Plasmid pRH195 underwent digestion with SalI and SpeI enzymes, resulting in plasmid linearization and excision  of the *XKS1* gene, forming pRH195d. The *Tr44175* gene was amplified from *T. reesei* cDNA using Tr44175_FW and Tr44175_Rv primers (Additional file 3: Table S2) while the *gfp* gene was amplified using GFP_Fw and GFP_Rv primers (Additional file 3: Table S2) from the pMCB17apx plasmid. pRH195d, along with *Tr44175* and *gfp* fragments, were transformed into SC9721 strain via the lithium acetate method [55]. The engineered *S. cerevisiae* strains were validated through PCR analysis and confocal microscopy. Subsequently, the Tr_44175_GFP strain was genetically transformed with either the pGH1-1 plasmid, containing the β-glucosidase-encoding gene *gh1-1* from *Neurospora crassa*, or the pBGL1B plasmid carrying the An03g03740 gene (*bgl1B*) (Additional file 4: Table S3) [26] which encodes a β-glucosidase from *Aspergillus niger*. Plasmid pBGL1B construction and An03g03740 gene synthesis was performed by *GenScript*. The Additional file 4: Table S3 provides descriptions of all the strains used and constructed in this work.

#### Confocal microscopy

Strains Tr44175_GFP were inoculated in plates containing medium YNB-trp with 20 g/L of glucose and incubated at 30 °C for 48 h. Then, isolated colonies were resuspended in 1 mL of PBS, centrifuged (14.000 rpm for 1 min), and washed two times using the same buffer. Posteriorly, the cells were resuspended in a final volume of 100 μL of 1X PBS and 10 μL of this solution was transferred to a glass slide. Each colony was observed on a Leica DMI6000 B Fluorescence Microscope, Germany, using a 100X oil-immersion objective lens equipped with an epifluorescence module and 100 W HBO mercury lamp. AxioCam camera (Carl Zeiss) was used to acquire differential Interference Contrast (DIC) and fluorescent images. The images were saved in TIFF format, and Adobe Photoshop 7.0 (Adobe Systems Incorporated, CA) was used to process the images.

#### Cultivation of *S. cerevisiae* strains on solid medium

For plate growth spot-assay, strains with pGH1-1 and p44175_GFP or pBGL1B and p44175_GFP were inoculated on solid YNB trp leu- (0,7 g/L YNB supplemented with 0.1 g/L lysine, 0.1 g/L uracil and 0.05 g/L histidine) or YNB trp ura- (0,7 g/L YBN supplemented with 0.1 g/L lysine, 0.1 g/L leucine and 0.05 g/L histidine) synthetic medium with 20 g/L glucose and incubated for 48 h at 30 °C. Colonies were collected, resuspended in carbon source-free medium, and incubated for 3 h to deplete internal carbon storage. After washing and centrifugation steps, cell pellets were resuspended in carbon source-free medium, and OD_600_ was measured. Serial dilutions were made (1:10), and 6 μL of each dilution were inoculated on specific media (YNB trp leu-synthetic medium, supplemented with 20 g/L of glucose, cellobiose (5, 10, 20 g/L), cellotriose (12 mg/L), cellotetraose (16 mg/L) or YNB trp ura- supplemented with glucose (20 g /L) or sophorose (8 mg/L, 16 mg/L, 24 mg/L). Yeast cells inoculated on media containing glucose or cellobiose, were incubated at 30 °C for 120 h, while those on media containing cellotriose, cellotetraose, or sophorose were incubated for 192 h. Posteriourly, the plates were photographed in the ChemiDoc XRS + System photodocumenter, Bio-Rad.

#### Growth of *S. cerevisiae* Strains in Liquid Medium

To confirm growth in different sugar concentrations, the Tr44175 growth assay was performed using cellobiose as sole carbon source in liquid media. Yeast strains were pre-grown in YNB medium supplemented with lysine, uracil, and histidine until reaching the stationary phase. Cells were washed with sterile water through centrifugation, then resuspended to an initial OD_600_ of 0.5 and introduced into carbon source-free synthetic medium. After 3 h of incubation, cultures were supplemented with 5, 10, or 20 g/L of cellobiose. Triplicate cultures were incubated for 144 h at 30 °C and 200 rpm. Samples were collected at specific time points for optical density measurement, microscopy analysis for contaminants, and HPLC analysis. All the experiments were performed in triplicate.

#### HLPC analysis

High-Performance Liquid Chromatography (HPLC) YL1900, coupled to a RID-10A refractive index detector (Alcrom model) was used to analyze cellobiose concentrations in the media. The separation occurred using a Rezex ROA-Organic Acid H+, Phenomenex column operated at 60 °C and eluted with 5 mM H2SO4 mobile phase at a flow rate of 0.5 mL/min. The YL-Clarity software was used to construct a cellobiose standard curve (5 mM to 100 mM) and to integrate the obtained data.

### Statistical analysis

The data were initially subjected to an analysis of variance (ANOVA) using Prism 9 software. Upon observing a significant difference, a post-hoc test was applied to identify significantly different means. Tukey test (α = 1%) was utilized to compare the means of growth curves and cellobiose consumption from yeast cell cultures. For RT-qPCR, the data were analyzed using a 1-way ANOVA followed by a Bonferroni post hoc test.

### Supplementary Information

**Additional file 1: Figure S1.** Tr4475 functional characterization.

**Additional file 2: Table S1.** Identification and reference of all the proteins used to construct the phylogenetic tree. The sugar transporters identified in this study are highlighted in red. The identification numbers can be searched in JGI Genome Portal (genome.jgi.doe.gov/portal) in the genomes: *Aspergillus nidulans*, *Aspergillus niger* NRRL3, *Neurospora crassa* clade B and *Trichoderma reesei* v2.0.

**Additional file 3: Table S2.** Primers used in this work.

**Additional file 4: Table S3.** Plasmids and strains used in this work.

## Data Availability

This manuscript contains all the data that was produced and examined during the study. An Additional File congaing supplementary information has also been provided.
