# Supplementary material for: Proteome profiling of enriched membrane-associated proteins unraveled a novel sophorose and cello-oligosaccharide transporter in Trichoderma reesei
Source: Microb Cell Fact. 2024 Jan 16;23:22. doi: 10.1186/s12934-023-02279-9 (PMC10790555; doi:10.1186/s12934-023-02279-9)
Supplement: Supplementary file 2 — Additional file 2: Table S1. Identification and reference of all the proteins used to construct the phylogenetic tree. The sugar transporters identified in this study are highlighted in red. The identification numbers can be searched in JGI Genome Portal (genome.jgi.doe.gov/portal) in the genomes: Aspergillus nidulans, Aspergillus niger NRRL3, Neurospora crassa clade B and Trichoderma reesei v2.0. [file 12934_2023_2279_MOESM2_ESM.docx]

**Table S1.** Identification and reference of all the proteins used to construct the phylogenetic tree. The sugar transporters identified in this study are highlighted in red. Distinct colors were employed to distinguish individual tree clades. The identification numbers can be searched in JGI Genome Portal (genome.jgi.doe.gov/portal) in the genomes: *Aspergillus nidulans*, *Aspergillus niger* NRRL3, *Neurospora crassa* clade B and *Trichoderma reesei* v2.0.

| **Clade A (Unkown - 38)** | **Ref.** | **Clade B (Mainly fructose - 37)** | **Ref.** | **Clade C  (Mainly glucose - 26)** | **Ref.** |
| --- | --- | --- | --- | --- | --- |
| Ncr197606 |  | Ang9728 |  | Ang7422 |  |
| Ncr69494 |  | And8262 |  | **Ncr445176** | Li et al., 2014 |
| Tre121441 |  | Ang379 |  | **Ncr490007** | Wang et al., 2017 |
| And4763 |  | Ncr512898 |  | Tre106556 |  |
| Ang4421 |  | **Tre123809** |  | And4453 |  |
| Ncr395478 |  | **Ang11807** | Coelho et al., 2013, de Vries et al., 2017 | **Ang5973** | de Vries et al., 2017 |
| **Tre44175** |  | **Ang3702** | Coelho et al., 2013 | **Ang8621** | de Vries et al., 2017 |
| Ang3935 |  | And9373 |  | **Tre62380** | Sloothaak et al., 2016 |
| **Tre80875** |  | Tre60945 |  | And1796 |  |
| Ang4846 |  | Ang3505 |  | And8742 |  |
| Ang11109 |  | And6978 |  | **Ang8911** | Sloothaak et al., 2015 |
| Ang9127 |  | And8234 |  | Ncr180834 |  |
| **Tre123702** |  | Ang3021 |  | **Ncr471140** | Wang et al., 2017 |
| Ncr493528 |  | And888 |  | **Tre47710** | Zhang et al., 2013, Wang et al., 2015, Zhang et al., 2015 |
| Tre82037 |  | Ang8232 |  | Ncr283880 |  |
| Ang7456 |  | Tre80767 |  | **Ang3147** | Vankuyk et al., 2004, Jørgensen et al., 2007 |
| And10348 |  | Ncr160226 |  | **And4727** | Dos Reis et al., 2013, Reis et al., 2016, Dos Reis et al., 2017 |
| Ang9931 |  | Ncr408824 |  | **And1082** | Forment et al., 2014, Dos Reis et al., 2013 |
| Ang5607 |  | Ncr47687 |  | **Ncr245379** | Olmedo et al., 2018 |
| Ang2859 |  | And4789 |  | Tre72383 |  |
| Tre60988 |  | Ang4386 |  | **And7483** | Dos Reis et al., 2013 |
| And9293 |  | And8901 |  | **Ang3879** | Sloothaak et al., 2015 |
| Ang10198 |  | Ang815 |  | **Tre63966** | Jiang et al., 2020 |
| Ang6839 |  | Tre80058 |  | Ncr405682 |  |
| Tre106118 |  | And10594 |  | **And3082** | dos Reis et al., 2013 |
| Ncr272876 |  | Ang11740 |  | **Ncr524132** | Wang et al., 2017 |
| Tre45868 |  | And3750 |  |  |  |
| Ang3274 |  | And8123 |  |  |  |
| And4366 |  | Tre26160 |  |  |  |
| Ang6060 |  | And4879 |  |  |  |
| Tre45852 |  | Tre105260 |  |  |  |
| And7278 |  | **Tre50618** |  |  |  |
| Ang7894 |  | And9287 |  |  |  |
| And6575 |  | **Ang5659** | de Vries et al., 2017 |  |  |
| Tre81389 |  | Tre75021 |  |  |  |
| Ang737 |  | Ncr157937 |  |  |  |
| Ncr395454 |  | **Tre68812** |  |  |  |
| Tre77552 |  |  |  |  |  |

**Table S1** (Continued).

| **Clade D  (Hexose, xylose - 54)** | **Ref.** | **Clade E  (Galactofuranose,  D-galacturonic acid - 40)** | **Ref.** | **Clade F (Xylose - 18)** | **Ref.** |
| --- | --- | --- | --- | --- | --- |
| And3117 |  | And10620 |  | Ang10134 |  |
| Ncr523194 |  | **Ang6866** | Arentshorst et al., 2020 | Ang1227 |  |
| Ang10141 |  | And1352 |  | Ang7442 |  |
| And5139 |  | Ang3902 |  | **Ang3272** | de Vries et al., 2017 |
| **Ang8653** | de Vries et al., 2017 | And9747 |  | And4182 |  |
| Tre27939 |  | Tre122013 |  | And8374 |  |
| Ang11406 |  | And5782 |  | **Ang9716** | Sloothaak et al., 2016 |
| And10341 |  | Ang2528 |  | **Tre121482** | Sloothaak et al., 2016 |
| Ang1360 |  | Ang1164 |  | And9693 |  |
| And2302 |  | And10633 |  | **Ang9364** | de Vries et al., 2017 |
| Ang3594 |  | Ang9077 |  | And1360 |  |
| Ang11786 |  | Ang4711 |  | **Ang1652** | de Vries et al., 2017 |
| Ang1270 |  | Ang10169 |  | And8873 |  |
| Ang7429 |  | Ang6805 |  | Tre54005 |  |
| And5018 |  | Ncr486548 |  | **Ang11715** | Sloothaak et al., 2016 |
| Ang4602 |  | **Tre65153** |  | **And6537** | Colabardini et al., 2014, Brown et al., 2016 |
| And3081 |  | And8887 |  | Ncr284711 |  |
| Ang11818 |  | Ang10300 |  | **Tre50894** | Sloothaak et al., 2016 |
| And9261 |  | And3524 |  |  |  |
| And6250 |  | **Ang958** | Sloothaak et al., 2014, Protzko et al., 2018 |  |  |
| Ang6305 |  | **Tre69026** | Protzko et al., 2018 |  |  |
| And6027 |  | Ang1651 |  |  |  |
| And1282 |  | And4930 |  |  |  |
| Tre48444 |  | Ang1110 |  |  |  |
| Ang11358 |  | And7558 |  |  |  |
| And8909 |  | Ang9845 |  |  |  |
| Ang834 |  | And1520 |  |  |  |
| Tre65191 |  | Tre77785 |  |  |  |
| Tre5656 |  | **Ncr511846** | Benz et al., 2014 |  |  |
| **Ncr433598** | Gao et al., 2017 | And10136 |  |  |  |
| Tre59388 |  | **Tre106330** | Protzko et al., 2018 |  |  |
| And3057 |  | And10346 |  |  |  |
| And4891 |  | And3769 |  |  |  |
| Tre65493 |  | **Tre53903** |  |  |  |
| Tre67541 |  | Ncr287920 |  |  |  |
| **Tre69957** | Nogueira et al., 2018 | Tre80091 |  |  |  |
| And9687 |  | And4881 |  |  |  |
| Tre55077 |  | Ang5631 |  |  |  |
| Tre109677 |  | And5558 |  |  |  |
| And10616 |  | Ang11036 |  |  |  |
| Ang7415 |  |  |  |  |  |
| Ncr507827 |  |  |  |  |  |
| And8390 |  |  |  |  |  |
| **Ang11710** | de Vries et al., 2017 |  |  |  |  |
| And2675 |  |  |  |  |  |
| Ncr509232 |  |  |  |  |  |
| Tre76758 |  |  |  |  |  |
| And2806 |  |  |  |  |  |
| And4915 |  |  |  |  |  |
| Tre67469 |  |  |  |  |  |
| And4390 |  |  |  |  |  |
| Ang6036 |  |  |  |  |  |
| Ncr515766 |  |  |  |  |  |
| Ncr397322 |  |  |  |  |  |

**Table S1** (Continued).

| **Clade G  (Xylose, Glucose, Hexoses - 21)** | **Ref.** | **Clade H  (Cellobiose, cellodextrin, lactose - 48)** | **Ref.** | **Clade I (Xylose - 51)** | **Ref.** | No Clade | **Ref.** |
| --- | --- | --- | --- | --- | --- | --- | --- |
| And545 |  | And535 |  | And9514 |  |  |  |
| Ang7294 |  | And5410 |  | Ang4329 |  |  |  |
| Tre60086 |  | Ang10866 |  | And9901 |  |  |  |
| Ang10305 |  | And54 |  | Ang5043 |  |  |  |
| And6452 |  | Ang5614 |  | Ncr424402 |  |  |  |
| Ang2090 |  | Ang5634 |  | **Tre121850** |  |  |  |
| Tre27770 |  | And9765 |  | Tre82309 |  |  |  |
| Tre61278 |  | Ang4244 |  | And579 |  |  |  |
| And5012 |  | And4449 |  | Ang4757 |  |  |  |
| And9165 |  | Ang3489 |  | Tre81670 |  |  |  |
| **Ncr480146** | Li et al., 2014, Li et al., 2015, Benz et al., 2014 Li et al., 2014, Li et al., 2015, Benz et al., 2014 | And8326 |  | Ncr413616 |  |  |  |
| **Tre104072** | Saloheimo et al., 2007 | And34 |  | And2695 |  |  |  |
| And8480 |  | Ang386 |  | Ang8798 |  |  |  |
| Ang8663 |  | Ncr143210 |  | Ang6252 |  |  |  |
| Tre69901 |  | Tre5890 |  | Tre69651 |  |  |  |
| And8466 |  | And5588 |  | Ang4325 |  |  |  |
| Ang6205 |  | Ang11054 |  | And9188 |  |  |  |
| **Tre22912** | Sloothaak et a., 2016 | And9022 |  | Ang5348 |  |  |  |
| **Ncr85956** | Gao et al., 2017 | Tre56684 |  | And9011 |  |  |  |
| And8235 |  | And7237 |  | Ang5552 |  |  |  |
| **Ang10052** | Sloothaak et al., 2016 | **Ang3028** | Lin et al., 2020 | Ang8841 |  |  |  |
|  |  | And4912 |  | And7582 |  |  |  |
|  |  | **Tre3405** | Ivanova et al., 2013, Zou et al., 2018 | And9769 |  |  |  |
|  |  | And8915 |  | And863 |  |  |  |
|  |  | **Ncr99563** | Ha et al., 2011 | Ang10202 |  |  |  |
|  |  | **Tre67752** | Casa-Villegas et al., 2018 | Ang2828 |  |  |  |
|  |  | **And9353** | Fekete et al., 2016, Reis et al., 2016, Havukainen et al., 2020 | And8486 |  |  |  |
|  |  | **Ncr99327** | Znameroski et al., 2014, Lin et al., 2017 | And8485 |  |  |  |
|  |  | Tre4774 |  | And8488 |  |  |  |
|  |  | Ang728 |  | **Ncr481400** | Du et al., 2010 |  |  |
|  |  | **And9631** | **Reis et al., 2016** | **Tre76800** |  |  |  |
|  |  | **Ncr465668** | Znameroski et al., 2014, Lin et al., 2017 | Ang817 |  |  |  |
|  |  | Ncr152297 |  | Ang935 |  |  |  |
|  |  | Tre77517 |  | And8246 |  |  |  |
|  |  | Ncr314017 |  | Ang235 |  |  |  |
|  |  | And6556 |  | Ncr403194 |  |  |  |
|  |  | Ncr82151 | Xiong et al., 2014, Li et al., 2015 | Ang10164 |  |  |  |
|  |  | Tre46819 |  | And7518 |  |  |  |
|  |  | And2121 |  | Ang7609 |  |  |  |
|  |  | And3092 |  | And1946 |  |  |  |
|  |  | Ang6836 |  | Ang2351 |  |  |  |
|  |  | Ang8396 |  | Tre62502 |  |  |  |
|  |  | And7005 |  | And6907 |  |  |  |
|  |  | Ang8009 |  | Ang4004 |  |  |  |
|  |  | And8405 |  | And78 |  |  |  |
|  |  | Ang1317 |  | Ang703 |  |  |  |
|  |  | And1553 |  | Ang4569 |  |  |  |
|  |  | Ang11470 |  | Ang5365 |  |  |  |
|  |  |  |  | Ang10175 |  |  |  |
|  |  |  |  | And8372 |  |  |  |
|  |  |  |  | Ang3213 |  |  |  |
