# Supplementary material for: Proteome profiling of enriched membrane-associated proteins unraveled a novel sophorose and cello-oligosaccharide transporter in Trichoderma reesei
Source: Microb Cell Fact. 2024 Jan 16;23:22. doi: 10.1186/s12934-023-02279-9 (PMC10790555; doi:10.1186/s12934-023-02279-9)
Supplement: Supplementary file 3 — Additional file 3: Table S2. Primers used in this work. [file 12934_2023_2279_MOESM3_ESM.docx]

**Table S2.** Primers used in this work.

| Primer ID | 5’ – 3’ sequence |
| --- | --- |
| Tr44175_Fw  Tr44175_Rv  GFP_Fw | AGTTCTTCTCCTTTACTCATTCCCCGTGTTCCTACCTCCTCAAC  GTTTTTTTAATTTTAATCAAAATGACGGCGGGC  GAATTAATAAAAGTGTTCGCTTAACGCCAAGCTTGCATGC |
| GFP_Rv | GGAACACGGGGAATGAGTAAAGGAGAAGAACTTTTCACTGG |
| GH1-1_Fw | GGCCTATCAGATTGAGGGT |
| GH1-1_Rv  Bgl1B_Fw  Bgl1B_Rv | CTTCTGGTCGTTCTCATA  ATGGGTTCTGCTACTGCTTC  TCTGGACCGATGAATTCACC |
