# Supplementary material for: Proteome profiling of enriched membrane-associated proteins unraveled a novel sophorose and cello-oligosaccharide transporter in Trichoderma reesei
Source: Microb Cell Fact. 2024 Jan 16;23:22. doi: 10.1186/s12934-023-02279-9 (PMC10790555; doi:10.1186/s12934-023-02279-9)
Supplement: Supplementary file 4 — Additional file 4: Table S3. Plasmids and strains used in this work. [file 12934_2023_2279_MOESM4_ESM.docx]

**Table S3.** Plasmids and strains used in this work.

| **Plasmid** | **Description** | **Reference** | |
| --- | --- | --- | --- |
| pRH195 | pBluescript II SK+, *TRP1, CEN6, ARSH4* + *PHXT7-XKS1-THXT7* | Hector et al., 2011 | |
| pGH1-1 | pRS425 PGK1p-gh1-1-CYC1t | Galazka et al., 2010 | |
| p44175_GFP | pRH195 +*Tr44175 +* GFP | This work | |
| pBGL1B | pSP-GM1 + *bgl1B* | This work | |
| **Strain** | **Genotype** | | **Reference** |
| SC9271 | MATa *his 3-D200 URA 3-52 leu2D1 lys 2D202 trp 1D63* | | *Fungal Genetic Stock Center* (FGSC) |
| Sc_Tr44175 | pTr44175-GFP, pBGL1B, SC9271 | | This work |
| Sc_Tr44175_BGL1B | pTr44175-GFP, pBGL1B, SC9271 | | This work |
| Sc_Tr44175_GH1-1 | pTr44175-GFP, pGH1 + SC9271 | | This work |
| Sc_pRH195m_pBGL1B  Sc_pRH195m_pGH1-1 | pRH195m, pBGL1B  pRH195m, pGH1 | | This work  This work |
