# Supplementary material for: Proteome profiling of enriched membrane-associated proteins unraveled a novel sophorose and cello-oligosaccharide transporter in Trichoderma reesei
Source: Microb Cell Fact. 2024 Jan 16;23:22. doi: 10.1186/s12934-023-02279-9 (PMC10790555; doi:10.1186/s12934-023-02279-9)
Supplement: Supplementary file 1 — Additional file 1: Figure S1. Tr4475 functional characterization. [file 12934_2023_2279_MOESM1_ESM.docx]

**Figure S1.** Tr4475 functional characterization. Growth of *S. cerevisiae* strain EBY_Tr44175::GFP in the presence of glucose 10g/L. The control strain contains the pRH195d empty vector. Maltose was used as a growth control. Yeast cells were incubated at 30 ºC for 7 days. To evaluate the Tr44175 capacity to transport glucose, the strain EBY.VW 4000, which is a hxt null strain incapable of growing in glucose, was utilized.
